# Medium-chain triglycerides may improve memory in non-demented older adults: a systematic review of randomized controlled trials

**DOI:** 10.1186/s12877-022-03521-6

**Published:** 2022-10-23

**Authors:** Panagiotis Giannos, Konstantinos Prokopidis, Irene Lidoriki, Konstantinos K. Triantafyllidis, Konstantinos S. Kechagias, Kamil Celoch, Darren G. Candow, Sergej M. Ostojic, Scott C. Forbes

**Affiliations:** 1 Society of Meta-Research and Biomedical Innovation, London, UK; 2 Department of Life Sciences, Faculty of Natural Sciences, Imperial College London, London, UK; 3 Department of Musculoskeletal Biology, Institute of Life Course and Medical Sciences, University of Liverpool, Liverpool, UK; 4 First Department of Surgery, National and Kapodistrian University of Athens, Laikon General Hospital, Athens, Greece; 5 Department of Nutrition and Dietetics, Musgrove Park Hospital, Taunton and Somerset NHS Foundation Trust, Taunton, UK; 6 Department of Metabolism, Digestion and Reproduction, Faculty of Medicine, Imperial College London, London, UK; 7 Department of Obstetrics and Gynaecology, Chelsea and Westminster Hospital NHS Foundation Trust, London, UK; 8 Department of Psychology and Neuroscience, Nova Southeastern University, Davie, USA; 9 Faculty of Kinesiology and Health Studies, University of Regina, Regina, Saskatchewan Canada; 10 Department of Nutrition and Public Health, University of Agder, Kristiansand, Norway; 11 Department of Physical Education Studies, Faculty of Education, Brandon University, Brandon, Manitoba Canada

**Keywords:** Medium-chain triglycerides, Nutritional ketosis, Cognitive function, Memory, Non-demented

## Abstract

**Background:**

Ketosis has been exploited for its neuroprotective impact and treatment of neurological conditions via ketone production. Exogenous medium-chain triglyceride (MCT) supplementation may induce nutritional ketosis. The aim of this systematic review is to explore the effects of MCTs on memory function in older adults without cognitive impairment.

**Methods:**

A systematic literature search of PubMed, Cochrane Library, Scopus, and Web of Science was employed from inception until April 2022 for randomized controlled trials (RCTs) in accordance with the Preferred Reporting Items for Systematic Reviews and Meta-Analyses (PRISMA) guidelines, investigating the impact of MCT oils on components of memory. Risk of bias (RoB2) tool was utilized for quality assessment.

**Results:**

Six trials were included for qualitative synthesis, in which two studies examined the effect of MCTs through a ketogenic meal. MCT supplementation compared to controls was associated with improved indices of memory function in 4 out of 6 studies, particularly working memory. A meta-analysis was not employed due to the low number of studies, therefore, a true effect measure of MCT supplementation was not explored.

**Conclusions:**

MCT supplementation may enhance working memory in non-demented older adults. These effects may be more prominent in individuals with lower baseline scores, from short and long-term supplementation. Further studies are warranted to confirm these findings in terms of optimal dose and MCTs composition, which may protect from memory decline during aging.

**Supplementary Information:**

The online version contains supplementary material available at 10.1186/s12877-022-03521-6.

## Introduction

Ketone bodies are an alternative energy substrate that can be utilized by brain cells under glucose-restricted circumstances such as prolonged fasting, exercise, or nutritional ketosis. Nutritional ketosis may be achieved through a ketogenic diet [1], exogenous ketone esters and salts [2], or medium-chain triglyceride (MCT) administration [3]. MCTs are absorbed and metabolized into ketone bodies via medium-chain fatty acids, which are comprised of β-hydroxybutyrate (BMB), acetoacetate, and acetone, and are metabolized in the liver through the portal vein, inducing ketone production [4]. Coconut and palm kernel oil are major sources of MCTs that are consumed either through diet or exogenous supplementation [5].

Nutritional ketosis has been utilized for its neuroprotective effects and treatment of neurological disorders. Specifically, ketogenic diets have been implemented in epilepsy [6] and Alzheimer’s disease [7], aiming to improve cognitive function. However, ketogenic dietary approaches could lead to malnutrition [8], therefore, exogenous ketone supplementation may be a convenient way to induce a ketogenic state [9]. Supplementation with ketone bodies can increase brain β-hydroxybutyrate (BHB) concentrations, enhancing mitochondrial biogenesis [10] and improving AD Assessment Scale-Cognitive Subscale (ADAS-cog) scores, processing speed, and memory in Alzheimer’s disease [11].

There are multiple memory systems in the human brain, each responsible for supporting distinct memory type. Emotional memory is responsible for ascribing emotional relevance to the experience, while declarative memory is linked to encoding events and facts. Additionally, motor memory is tied to the ability to execute actions and action sequences, and working memory is temporarily holding onto information. Whilst the exact neurobiology underpinning memory processes is beyond the scope of this article, the literature is clear as to the role of glucose in allowing the brain to meet its metabolic demands [12]. Indeed, glucose is thought to have a facilitating effect on cognitive functioning due to the brain’s reliance of glucose as its primary fuel, a phenomenon known as ‘glucose memory facilitation effect’ [13].

Human brain is the most energetically demanding organ, accounting for over 20% of total body energy usage while comprising only approximately 2% of its weight [14]. The fundamental units of the nervous system that are neurons, communicate electrically via the process of neurotransmission, a process that is energetically costly for the brain to perform and susceptible to disruptions that might entail metabolic perturbations. As a result, the brain possesses an exquisitely delicate mechanism for ensuring adequate fuel availability [15].

Various neurological conditions are associated with a deficit in brain energy metabolism, oftentimes characterized by acute or chronic glucose hypometabolism [15]. The theoretical effect of MCT stems from their ability to provide energy supply to the brain [16], especially during states of brain energy metabolic crisis [15]. Investigations have also been undertaken in order to determine whether MCT ingestion would improve some aspects of cognitive function in young and healthy populations, as measured by a standardised battery of laboratory-based cognitive tests including trial making, digit and working memory span, covert shift of attention, and rapid visual information processing (sustained attention) [17].

Although research has revealed a positive impact of MCT oils on several measures of cognitive function in young individuals and older populations with neurological disorders, the effects of MCT oil supplementation on indices of cognition in older individuals without cognitive impairment, remain largely unexplored. Therefore, the aim of this systematic review was to investigate the potential effects of MCT oil supplementation on measures of memory function in non-demented older populations.

## Methods

This systematic review was conducted in accordance with the Preferred Reporting Items for Systematic Reviews and Meta-Analyses (PRISMA) guidelines (Page 2021). The protocol was registered in the International Prospective Register of Systematic Reviews (PROSPERO) (CRD42022304319).

### Search strategy

Two independent reviewers (KP and KKT) searched PubMed, Scopus, Web of Science, and Cochrane library from inception until April 2022. A search strategy involving the following terms was used: “medium-chain triglycerides” OR “MCT oils” AND “cogn*” OR “memory”. The comprehensive search strategy used is described in the supplementary materials (Table S1). A manual search of references cited in the selected articles and published reviews was also performed. Discrepancies in the literature search process were resolved by a third investigator (PG). Studies were included based on the following criteria: (1) must be a randomized controlled trial; (2) included participants with a mean of 60 years and above without cognitive impairment; (3) the intervention group received MCT oil supplementation; (4) the control group received a placebo or appropriate non-placebo treatment; (5) assessed cognitive performance outcomes (Table S2). Studies were excluded if they: (1) were non-clinical trials; (2) included participants with cognitive impairment; (3)a full text was not available.

### Data extraction

Two authors (KP and KKT) extracted data regarding the date of publication, study design, participant health status and sample size, age, sex, measures of cognitive function, and treatment form, dose, and duration. Disagreements between authors were resolved by a third and fourth reviewer (PG, KSK).

### Quality assessment of studies

The quality of included studies was assessed using the Cochrane Risk-of-bias 2 (RoB2) tool and evaluated by three independent reviewers (KP, PG, KKT). Appraisal of bias risk using the RoB2 tool included assessment of the domains of bias in randomized clinical trials: randomization process; deviations from intended interventions; missing outcome data; measurement of the outcome; selection of the reported result. According to the RoB2 tool scoring system, study quality was defined as low, some concerns, or high risk of bias.

## Results

### Search results

**Fig. 1:**
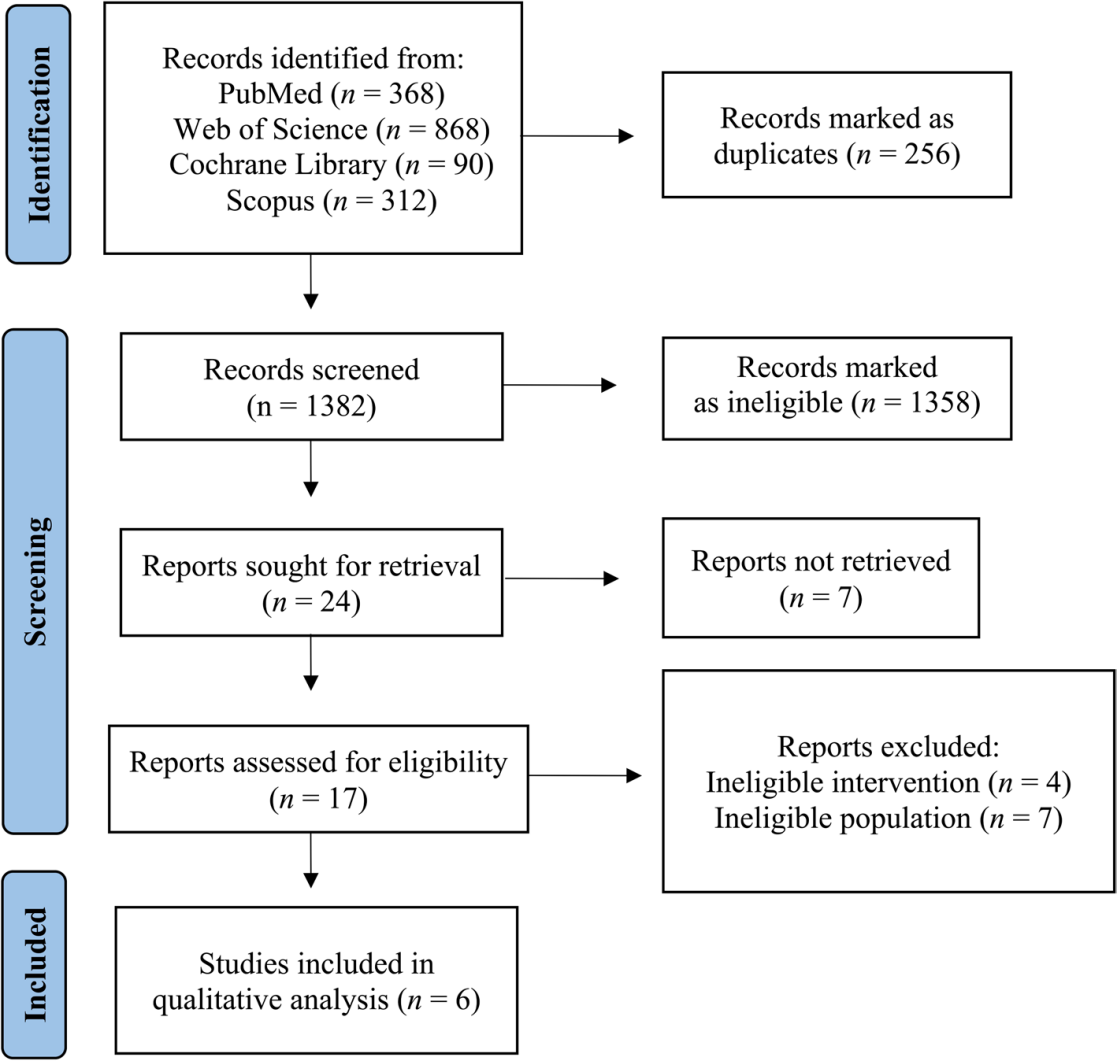
Flowchart of the employed literature search

The initial literature search tallied 1638 publications. After the exclusion of 256 duplicates, 1382 unique publications were screened. Overall, 1358 publications were marked as ineligible due to irrelevant study design and study population. Full-text screening of the remaining 24 publications resulted in 17 eligible RCTs examining MCT oil supplementation on measures of cognitive performance. Of these, seven studies had ineligible study population and four had ineligible intervention. Six RCTs were included in the systematic review (Fig. 1) [16, 18–22].

### Characteristics of the included studies

Four studies used MCT oils as a supplement [18–20, 22], while two studies used MCT oils in a mixed meal [16, 21]. MCT supplementation was given daily in four studies[18–20, 22]and on separate days in two studies [16, 21]. Treatment duration ranged from < 10 days [16, 21], two weeks [19], to three months [18, 20, 22]. Four studies were double-blind [16, 19, 21, 22], from which two were crossover studies [16, 21], and two studies were single-blind [18, 20]. A comprehensive description of the included studies is presented in Table 1.

**Table 1:** Study and participant characteristics of the included studies. Data are expressed as mean (standard deviation), unless otherwise stated

Study, year	Study design	Total	MCT		Control		Treatment dose (g/d)	Treatment duration	Control	Health status	Memory Outcomes
		***n*** **(M/F)**	***n*** **(M/F)**	**Age**	***n*** **(M/F)**	**Age**					
Mutoh, 2022 [22]	Double-blind RCT	63	32 (10/22)	69.8 (4.0)	31 (10/21)	70.3 (4.1)	18	3 months	Placebo (LCT)	Healthy	-LM-IA -LM-IB -LM-IIA -LM-IIB (immediate & delayed logical memory)
Yomogida, 2021 [21]	Double-blind crossover RCT	20 (6/14)	20 (6/14)	65.7 (3.9)	15 (13/2)	61 (9.4)	19.9^a^	6.9 (0.3) days	Placebo (LCT)	Healthy	-Working memory (N-back test)
Abe, 2020 [20]	Single-blind RCT	31	16	85.5 (6.8) All	15	85.5 (6.8) All	6	3 months	Placebo (LCT)	Nursing home residents	-MMSE (memory recall) -NM (logical memory)
O’neill, 2019 [19]	Double-blind RCT	80	38	65.4 (6.2)	42	65.4 (6.2)	10, 20, 30, or 40	2 weeks	Placebo	Healthy	-Immediate and delayed memory (VRM task) -Spatial working memory (SWM task)
Abe, 2017 [18]	Single-blind RCT	25 (7/18)	13 (4/9)	85.5 (3.7)	12 (3/9)	86.8 (6.5)	6 & 1.2 g leucine & 20 μg cholecalciferol	3 months	LCT & 1.2 g leucine & 20 μg cholecalciferol	Nursing home residents	-MMSE (memory recall) -NM (logical memory)
Ota, 2016 [16]	Double-blind crossover RCT	19 (6/13)	19 (6/13)	66.1 (2.9)	19 (6/13)	66.1 (2.9)	20^a^	9.5 (6.9) days	Placebo	Healthy	-Working memory (Digit span test) -Visual memory (visual memory span test) -Short-term memory (letter-number sequencing test)

*LCT* Long-chain triglycerides, *LM-I* Logical memory-immediate recall, *LM-II* Logical memory-delayed recall, *MCT* Medium-chain triglycerides, *MMSE* Mini-mental state exam, *NM* Nishimura geriatric rating scale for mental status, *RCT* Randomised controlled trial, *SWM* Spatial working memory, *VRM* Verbal recognition memory

^a^Data were collected in two separate visits and not daily

### Memory outcomes and definitions

Four studies measured overall working memory, using the spatial working memory (SWM) task [19], N-back [21], and Digit and Spatial Span Tests [16]. Two studies tested immediate memory recall using a mini-mental state exam (MMSE) [18, 20], while one study measured immediate and delayed memory using the verbal recognition memory (VRM) task [19]. Logical memory was evaluated via the Nishimura geriatric rating scale for mental status (NM) [18, 20] and via the Wechsler Memory Scale-Revised test through immediate recall (LM-I) and delayed recall (LM-II) [22]. Finally, one study measured visual memory through visual memory span test and short-term memory through a letter-number sequencing test [16].

The SWM task examines the ability to remember and modify spatial information, in which participants search for blue tokens from multiple squares on a screen. After a blue token is found, participants should find the next token that is not being hidden in the previous box.

During the N-back test, participants are required to press a button each time they see the letter “X”, defined as the “0-back” condition. In addition, participants need to press the button in which the letter they saw was identical to the letter that was observed two letters before, known as the “2-back” condition.

The VRM task examines the immediate and delayed memory of verbal information. Participants are presented with a list of 12 words -one at a time- and are required to produce the maximum number of possible words associated with this word, recognizing target words from the list of target and distracter words.

The Digit Span test assess the ability of repeating a series of numbers backwards, while the Spatial Span test examines the ability of tapping blocks (forwards or backwards) in a certain predetermined order.

MMSE is a 30-point test comprised of multiple measures of cognitive function, including memory. In MMSE, memory is assessed through a series of questions regarding word repeating (forwards and backwards) and remembering the number of objects presented a few minutes prior to the question.

The NM scale is another tool used for the measurement of overall cognitive performance. Memory function using the NM scale is measured on a 0–5 scale, in which participants are asked to recall a story. “No memory” is represented by the score of zero, while five represents a good overall memory function.

For the assessment of LM-I and LM-II, participants listened to two short stories who were then instructed to provide comprehensive details immediately and after 30 min.

Short-term memory was assessed via letter-number sequencing test, which measures the participants’ ability to process and re-sequence information.

### Risk of bias assessment

**Fig. 2:**
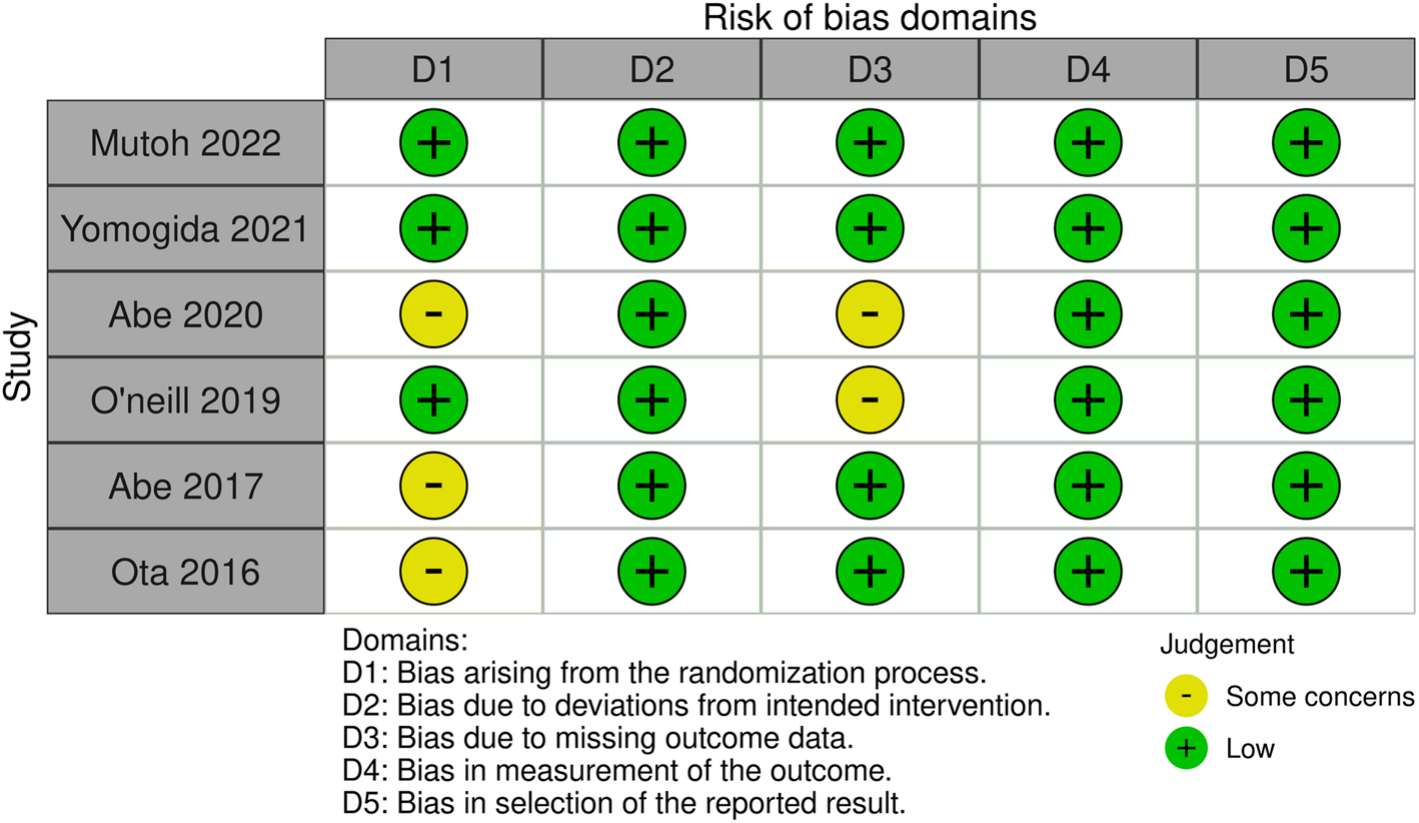
Risk of bias assessment of the included studies

Of the included studies, three RCTs [16, 18, 20] had some concerns considering the lack of information on how allocation concealment was performed. Furthermore, two RCTs [19, 20] had some concerns given the increased drop-out rate, whereas the remaining studies had an overall low risk of bias. A traffic light plot is presented in Fig. 2.

### Memory

No significant changes for working memory were found using the N-back test following a 19.9 g MCT meal compared to placebo during two separate visits, one week apart (MCT: 77.86 ± 2.99; Placebo: 73.39 ± 3.57) [21].

Evaluation of memory recall via MMSE revealed a significant increase following 6 g/d of MCT for 3 months (pre: 17.5, 95% CI 14.9 – 20.2; post: 21.0, 95% CI 18.3 – 23.7) compared to an isocaloric long chain triglyceride (LCT) supplement (pre: 17.0, 95% CI 14.4 – 19.6; post: 16.3, 95% CI 13.6 – 18.9) [20].

Furthermore, memory function using the NM scale revealed a higher score in the intervention group (pre: 6.2, 95% CI 5.2 – 7.1; post: 7.1, 95% CI 6.1 – 8.1) compared to the control group (pre: 6.6, 95% CI 5.7 – 7.6; post: 5.7, 95% CI 4.7 – 6.7). The same authors conducted a similar study, in which they added 1.2 g/d leucine and 20 μg/d cholecalciferol in both the MCT and LCT groups. Similarly, no significant changes were found in memory recall after 3 months (MCT: pre: 1.3 ± 1.3, post: 1.5 ± 1.3; LCT: pre: 1.8 ± 1.4, post: 1.7 ± 1.4), while NM scale memory function demonstrated an increased score in the MCT (pre: 4.7 ± 2.4, post: 6.7 ± 2.0) compared to LCT (pre: 7.1 ± 3.4, post: 5.8 ± 3.3). In addition, no changes were observed in immediate (LM-IA; MCT: pre: 11.3 ± 4.1, post: 14.1 ± 4.1; Placebo: pre: 13.3 ± 3.5, post: 14.9 ± 3.8 – LM-IB;MCT: pre: 9.5 ± 3.1, post: 10.8 ± 3.5; Placebo: pre: 10.4 ± 3.5, post: 12.1 ± 2.9) and delayed recall (LM-IIA; MCT: pre: 8.7 ± 4.6, post: 11.2 ± 5.1; Placebo: pre: 10.0 ± 3.8, post: 12.2 ± 4.1 – LM-IIB; MCT: pre: 7.5 ± 3.4, post: 9.9 ± 3.8; Placebo: pre: 8.3 ± 3.7, post: 10.2 ± 3.9) after 3 months [22].

Moreover, no changes were seen between the MCT (40 g) and placebo after 2 weeks in immediate (MCT: -0.06; placebo: -0.37) and delayed recall (MCT: -2.54; placebo -1.98) using the VRM task [19]. Spatial working memory (SWM task) changes were also similar in the MCT (pre: -0.96, post: -1.96) and placebo groups (pre: -0.29, post -1.83) [19]. Working memory measured through a digit span test showed no changes following a 20 g MCT meal on two separate occasions compared to placebo (20 g LCT) in both 90 min (MCT: 9.5 ± 2.7; placebo: 8.6 ± 3) and 180 min conditions (MCT: 9.9 ± 2.9; placebo: 9.6 ± 3) [19].

Finally, visual memory span was also similar in both the 90 min (MCT: 12.2 ± 2.2; LCT: 11.7 ± 3.1) and 180 min conditions (MCT: 12.2 ± 2.3; LCT: 12 ± 2) [16]. Effects in number and the statistical significance observed between the intervention and control groups are detailed in Table 2.

**Table 2:** Intervention, control, and intragroup effects and statistical significance within included studies. Data are expressed as mean (standard deviation), unless otherwise stated

Study, year	Outcome	Control group			Intervention group			Intergroup *P*-value
		**Pre-intervention, *n***	**Post-intervention, *n***	***P*-value**	**Pre-intervention, *n***	**Post-intervention, *n***	***P-*value**	
Mutoh,2022 [22]	LM-IA	13.3 (3.5)	14.9 (3.8)	0.003	11.3 (4.1)	14.1 (4.1)	< 0.001	0.078
	LM-IB	10.4 (3.5)	12.1 (2.9)	0.002	9.5 (3.1)	10.8 (3.5)	0.013	0.146
	LM-IIA	10 (3.8)	12.2 (4.1)	< 0.001	8.7 (4.6)	11.2 (5.1)	< 0.001	0.285
	LM-IIB	8.3 (3.7)	10.2 (3.9)	< 0.001	7.5 (3.4)	9.9 (3.8)	< 0.001	0.552
Yomogida, 2021 [21]	Working memory (N-back test)	NA	73.39 (3.57)	NA	NA	77.86	NA	NA
Abe, 2020 [20]	MMSE	17	16.3	NA	17.5	21	NA	NA
	NM	30.9	27.5	NA	31	33.7	NA	
O’neill, 2019 [19]	VRM task	NA	NA	NA	NA	NA	NA	NA
	SWM task	NA	NA	NA	NA	NA	NA	NA
Abe, 2017 [18]	MMSE	1.8 (1.4)	1.7 (1.4)	NA	1.3 (1.3)	1.5 (1.3)	NA	NA
	NM	7.1 (2.8)	6 (3.3)	< 0.05	4.7 (2.4)	6.7 (2)	< 0.001	0.001
Ota, 2016 [16]	Digit-span test	NA	9.6 (3)	NA	NA	9.9 (2.9)	NA	0.41
	Visual memory span test	NA	12 (2)	NA	NA	12.2 (2.3)	NA	0.64
	Letter-number sequencing test	NA	11.8 (2.3)	NA	NA	11.8 (1.6)	NA	0.75

*LM-I* Logical memory-immediate recall, *LM-II* Logical memory-delayed recall, *MMSE* Mini-mental state exam, *NA* Not available, *NM* Nishimura geriatric rating scale for mental status, *SWM* Spatial working memory, *VRM* Verbal recognition memory

## Discussion

MCTs have garnered considerable attention as an effective dietary strategy to improve cognitive function in patients with neurological disorders [15, 23], however, less is known regarding the potential beneficial effects of MCT supplementation in individuals without cognitive impairment. MCTs are converted to ketone bodies that, in turn, constitute an alternative source of energy for neurons. Hyperketonemia induced by MCT intake might be the main cause of acute and chronic changes in several cognitive functions [24]. The aim of the present systematic review of randomized controlled trials was to investigate whether these effects attributed to MCT supplementation could be extended to older individuals without cognitive deficits.

According to our results, MCT oil supplementation was associated with better cognitive outcomes in 4 out of 6 studies. More specifically, two studies examined the effect of MCT oils via the ingestion of a single ketogenic meal [16, 21] on cognitive function of older non-demented adults.Performance on working memory was significantly better 90 min after 19.9 g of MCT included in a mixed meal compared to placebo (N-back task) [21]. In addition, improvements in working memory, visual attention, and task switching after 90 and 180 min following oral intake of 20 g of MCTs were displayed, however, these changes were not statistically different compared to placebo [16].

Another two studies found significant positive associations between MCT oil supplementation and cognitive function in elderly individuals residing in nursing homes [18, 20]. MCT supplementation (6 g/d) in combination with 1.2 g/d leucine and 20 μg/d cholecalciferol for three months was effective at increasing memory function by 30.6% and 10.6% as evaluated by MMSE and NM scale respectively, as opposed to control using LCT that displayed a reduction NM scale and no changes in MMSE scores [18]. The same research team conducted a randomized controlled trial in which the intervention group received daily MCT supplementation (6 g) for three months. Similar to the first study, MCT oil intake was associated with a significant increase in the MMSE scores at three months, while a significant difference in MMSE score between the MCT and LCT groups was also observed. However, MMSE scores returned to baseline values at the 4.5-month post-intervention follow-up visit [20], posing a crucial question concerning the impact of duration of MCT supplementation.

Nevertheless, the effect of MCT oils on selected parameters of cognitive function appear to be altered by baseline scores in cognitive assessment [16, 21]. For instance, subgroup analysis revealed that participants with higher global cognitive function had better performance in the working memory task after MCT supplementation, while participants with relatively lower cognitive function exhibited positive changes in the inhibitory control task [21]. In accordance with the previous finding, Ota et al. found a significant effect of a ketogenic meal mainly in individuals who had lower baseline global scores in the evaluation of integrative cognitive function [16]. One possible explanation is that a ketogenic meal could only affect individuals with impaired glucose metabolism accompanied by a mild level of age-related cognitive decline. Indeed, a negative association between cerebral glucose metabolism and ageing has been observed [25]. Furthermore, the differences seen only in some types of cognitive assessment could be ascribed to the fact that several procedures during cognitive testing are more demanding, meaning there is greater capacity for improvement.

MCTs either in the form of an oral supplement or added to regular meals, are rapidly absorbed by the portal system and beta-oxidized in the liver, generating excess acetyl-CoA, which forms ketone bodies. Chronic MCT ingestion may improve cognitive performance through metabolic adaptations observed in brain cells, mainly associated with increased number of mitochondria, enhanced mitochondrial function and reduced mitochondrial oxidative stress [26, 27]. Furthermore, MCTs possess immune modulating properties [28] and alters satiety, energy expenditure, and body composition, which may influence cognition [29]. In addition, a higher ketogenic effect is observed when MCTs are consumed without a concomitant meal which contains a substantial quantity of carbohydrates [30], supporting the notion that as the amount of carbohydrate consumed with the MCTs increases, the ketogenic response decreases. However, it is important to note that glycogen and glucose metabolism plays a critical role in astrocytes and long-term memory formation [31]. Contrary to the aforementioned results, a recent study concluded that supplemental daily intake of 30 g GSK2981710, a MCT, for 14 days did not result in significant changes in memory function in older adults 55–80 years of age [19]. Likewise, another recent study providing MCT supplementation (18 g/d) after 3 months did not elicit beneficial responses in immediate and delayed logical memory in healthy older adults compared with placebo [22]. Of note, composition of MCTs should be considered when examining the magnitude of their effects. Particularly, the effects of MCTs on cognitive capabilities may be affected by caprylic acid (C8) to capric acid (C10) ratio. Caprylic acid (C8) induces astrocyte ketogenesis to a greater extent compared to capric acid (C10) and is associated with elevated ketone bodies [26, 32, 33]. Both C8 and C10 can cross the blood brain barrier [34, 35]. In the mitochondria, C8 and C10 are oxidized into acetyl-CoA, which in turn can enter the citric acid cycle further supporting cellular metabolism. MCTs promote astrocyte glutamine synthesis, with relevant data supporting that C8 outperforms C10 regarding cellular metabolism in the brain [36]. However, these findings remain controversial [37] and more research is warranted [38]. Moreover, notwithstanding the high rates of compliance with MCT supplementation observed in clinical trials, gastrointestinal symptoms, such as diarrhea, intestinal gas, nausea, and vomiting have been reported. These side effects are more commonly reported with C8 supplementation, potentially leading to lower adherence [39].

The present review is, to our knowledge, the first systematic review of randomized clinical trials investigating MCT supplementation on cognitive function in older adults without cognitive impairment. However, there are also some limitations that need to be addressed. First, the assessment of participants’ memory function was based on different tools. Second, MCT dose, specific composition, and treatment duration differed between studies, potentially confounding the results. Moreover, the presence of an ApoE4 allele, known to modulate cognition in response to MCTs, was not examined in any of the included studies. Based on the aforementioned limitations, a meta-analysis was not able to be conducted at present. Additionally, it is important to highlight that MCT may have a more profound effect on cognition when the brain is stressed (i.e., seizure disorders, mild cognitive impairment, Alzheimer’s disease, and neurotrauma) and may be of interest to tactical personnel particularly during hypoxia [15], however future research is required. Lastly, the clinical significance of MCT supplementation regarding memory function needs to be addressed in future trials.

## Conclusions

Collectively, MCT supplementation was significantly associated with better memory outcomes, specifically regarding working memory in non-demented older adults. This finding was more robust in participants with lower baseline scores in memory tasks even with short-term supplementation. Despite these promising findings, more research is warranted to confirm these findings, as well as to specify the optimal dose and composition of MCT oils related to long term improvement of cognitive function and protection from neurodegenerative diseases accruing during aging.

## Supplementary Information

**Additional file 1: Table S1. **Search terms employed in the literature search.

**Additional file 2: Table S2.** Population, Intervention, Comparison, Outcomes and Study (PICOS) criteria for the inclusion of studies in the systematic review.

## Data Availability

All data generated or analysed during this study are included in this published article.
