# Supplementary material for: Medium-chain triglycerides may improve memory in non-demented older adults: a systematic review of randomized controlled trials
Source: BMC Geriatr. 2022 Oct 23;22:817. doi: 10.1186/s12877-022-03521-6 (PMC9588230; doi:10.1186/s12877-022-03521-6)
Supplement: Supplementary file 1 — Additional file 1: Table S1. Search terms employed in the literature search. [file 12877_2022_3521_MOESM1_ESM.docx]

**Table S1.**Search terms employed in the literature search.

| **Database** | **Search terms** |
| --- | --- |
| PubMed | ((Medium Chain Triglycerides OR MCTs OR coconut oil OR palm kernel oil) AND (cogn* OR digit span OR recall test OR attention OR stroop test OR psychomotor speed OR reaction time OR flanker task OR executive function OR memory)) |
| Cochrane Library | ((Medium Chain Triglycerides OR MCTs OR coconut oil OR palm kernel oil) AND (cogn* OR digit span OR recall test OR attention OR stroop test OR psychomotor speed OR reaction time OR flanker task OR executive function OR memory)) |
| Web of Science | TS= ((Medium Chain Triglycerides OR MCTs OR coconut oil OR palm kernel oil) AND (cogn* OR digit span OR recall test OR attention OR stroop test OR psychomotor speed OR reaction time OR flanker task OR executive function OR memory)) |
| Scopus | TITLE-ABS-KEY ((Medium Chain Triglycerides OR MCTs OR coconut oil OR palm kernel oil) AND (cogn* OR digit span OR recall test OR attention OR stroop test OR psychomotor speed OR reaction time OR flanker task OR executive function OR memory)) |
