# Supplementary material for: Medium-chain triglycerides may improve memory in non-demented older adults: a systematic review of randomized controlled trials
Source: BMC Geriatr. 2022 Oct 23;22:817. doi: 10.1186/s12877-022-03521-6 (PMC9588230; doi:10.1186/s12877-022-03521-6)
Supplement: Supplementary file 2 — Additional file 2: Table S2. Population, Intervention, Comparison, Outcomes and Study (PICOS) criteria for the inclusion of studies in the systematic review. [file 12877_2022_3521_MOESM2_ESM.docx]

**Table S2.** Population, Intervention, Comparison, Outcomes and Study (PICOS) criteria for the inclusion of studies in the systematic review.

| **P**articipants | Non-demented people ≥ 60 years of age |
| --- | --- |
| **I**ntervention | MCT oil supplementation |
| **C**omparison | Placebo or appropriate non-placebo treatment |
| **O**utcomes | Indices of memory function |
| **S**tudy design | Randomized controlled trial |

MCT, medium-chain triglycerides
